# Designing Multimodal Interactive Dashboard of Disaster Management Systems

**DOI:** 10.3390/s22114292

**Published:** 2022-06-05

**Authors:** Abeer AlAbdulaali, Amna Asif, Shaheen Khatoon, Majed Alshamari

**Affiliations:** 1 Information Systems Department, College of Computer Sciences and Information Technology (CCSIT), King Faisal University, P.O. Box 400, Al-Ahsa 31982, Saudi Arabia; 219002461@student.kfu.edu.sa (A.A.); smajed@kfu.edu.sa (M.A.); 2 School of AI and Advanced Computing, Xi’an Jiaotong-Liverpool University, Suzhou 215000, China; shaheen.khatoon@xjtlu.edu.cn

**Keywords:** emergency management, interactive visualization, social media multimodal information, dashboard, usability and user experience

## Abstract

Disasters and crises are inevitable in this world. In the aftermath of a disaster, a society’s overall growth, resources, and economy are greatly affected as they cause damages from minor to huge proportions. Around the world, countries are interested in improving their emergency decision-making. The institutions are paying attention to collecting different types of data related to crisis information from various resources, including social media, to improve their emergency response. Previous efforts have focused on collecting, extracting, and classifying crisis data from text, audio, video, or files; however, the development of user-friendly multimodal disaster data dashboards to support human-to-system interactions during an emergency response has received little attention. Our paper seeks to fill this gap by proposing usable designs of interactive dashboards to present multimodal disaster information. For this purpose, we first investigated social media data and metadata for the required elicitation and analysis purposes. These requirements are then used to develop interactive multimodal dashboards to present complex disaster information in a usable manner. To validate our multimodal dashboard designs, we have conducted a heuristic evaluation. Experts have evaluated the interactive disaster dashboards using a customized set of heuristics. The overall assessment showed positive feedback from the evaluators. The proposed interactive multimodal dashboards complement the existing techniques of collecting textual, image, audio, and video emergency information and their classifications for usable presentation. The contribution will help the emergency response personnel in terms of useful information and observations for prompt responses to avoid significant damage.

## 1. Introduction

### 1.1. Disaster Management Systems

Emergency management (EM) is the responsibility of multiple workforces that do not work together routinely (e.g., firefighters, police, and medical providers) [1]. The EDM (emergency decision-making) helps humanitarian organizations with emergency and rescue operations during disasters [2]. The command and control centers (C&CC) help coordinate activities for diverse workforces in order to enhance the quality of the emergency response, which reflects the amount of thinking done in advance by the emergency responders. Consequently, by quickening the emergency response, much damage can be controlled, and sometimes, responders can prevent the emergency from becoming a disaster. Satellite data [2,3] are a traditional source of collecting disaster information from the crisis place; however, in recent years, there has evidently been a massive revolution in EDM, and researchers are starting to look for other available resources for quick retrieval of disaster-related information, and a popular source is social media [4]. Social media information has proven to be a very useful source in EM in all phases of crisis (i.e., stage 1 is the calm before the storm; stage 2 is the storm; stage 3 is the peak; stage 4 is the plateau; stage 5 is the decline; and stage 6 represents a return to normal [5]). Moreover, social media gives access to such information, as it is posted directly by the people at the disaster site. At present, social media is a source of diverse and multimodal information that comes directly from users, and its use is indispensable for accessing information on natural and man-made disasters.

In the past, several studies have been conducted on capturing useful disaster-related information from social media text [6], such as disaster topic classification [7,8,9,10], audio–video event detection [8], image detection, and classification algorithms for image-based crisis event detection [11,12,13]. The previous studies are interesting in that the social media data (text, images, audio, and video) were not only used to detect the disaster, but they were further preprocessed and modeled to classify the severity and nature of the event. Such data are significantly helpful in identifying the context and exact nature of the incident to identify its emergency response needs. Not presenting the data to the right stakeholders in a usable way is counterproductive. Dashboards have received trivial attention in terms of the usable representation of such information. Furthermore, not much research has been conducted on how interactive dashboards can represent multimodal crisis-related data.

This research paper aims to present heterogeneous disaster information using interactive dashboards, and it is driven by the previous research contributions in textual, audio, and visual data collection, as well as incident extraction for an efficient emergency response. It is undeniable that intelligent detection, identification, and classification of disaster information plays a significant role in disaster management systems. Moreover, we cannot negate the significance of presenting this information on interactive dashboards in a usable manner to responsible bodies, in order to support their humanitarian emergency and rescue efforts.

### 1.2. The Objectives of Developing Multimodal Interactive Dashboards of Disaster Management Systems

The previous studies lacked contributions in multimodal disaster information presentation and visualization to monitor disaster information efficiently. In this paper, we applied a user-centered design approach for developing the designs of an interactive dashboard for the presentation of heterogeneous data related to different types of disasters, but mainly natural disasters. The objectives of our research are:

- To explore the multimodal (text, image, audio, and video) data requirements for developing interactive dashboards for displaying disaster-related information.
- To research ways to integrate text, image, audio, and video information to create multimodal interactive disaster dashboards.
- To design and prototype the interactive disaster dashboards using the user-centered design approach.
- To evaluate the prototypes of disaster-related interactive dashboards using usability evaluation techniques.

In the remainder of this paper, we report the related work in Section 2, we identify the motivation of our contribution in Section 3, explain our methodology in Section 4, present our proposed design and report the evaluation results in Section 5, and close our paper in Section 6 with a discussion and conclusion.

## 2. Related Work

Several researchers proposed methods and tools to enhance social media data analysis during crisis events, and they offered some data visualization techniques to present the results. Chae et al. [14] investigated people’s movement patterns during a crisis by monitoring Twitter posts during Hurricane Sandy and a short-term tornado. Their methodology consisted of multiple analysis methods, including interactive spatiotemporal visualization. The collected data was visualized using a heatmap that helped analysts understand how users react to different events by comparing the spatial-temporal patterns during unusual circumstances. Kwon and Kang [15] proposed utilizing social media data to detect signs of disasters before they happen. In their study, they targeted flood damage. After extracting and filtering flood-related tweets that included geolocations, these tweets were then classified using a 5-by-5 risk evaluation matrix that was displayed on a map as points, and each point was colored based on the risk level. These studies prove that effective visualization of data is essential to benefit from big data computing results; however, these studies do not visualize multiple data types using multiple visualization techniques. The presented studies contributed to the area of textual social media disaster data presentation, but multimodal disaster data presentation has not been investigated.

Onorati et al. [16] developed a visualization tool that helps improve the decision-making process for emergency operators. They carried out an exploratory study with 20 emergency management experts to investigate what social media data is needed and how they can be best presented. Results show that geolocation data and tools for searching and filtering are highly valued. The visualization tool used four techniques: treemaps, word clouds, bubble charts, and an animated map. The authors observed that including different visualization techniques were beneficial when participants used mixed methods to complete the tasks. Although they presented the data using multiple visualization techniques, they focused only on textual data. Similarly, some studies in the literature have attempted to visualize image data. A study by Bhargava et al. [17] suggested using image treemaps and image spaces (scatterplots). The use of treemaps is an approach of displaying information whereby quantitative data determine the relative physical space that a data-point takes up. For example, to create a treemap of images, that space is filled with the image instead of a color. The study used image treemaps to show the top 30 stories covered by news channels, which were then sorted and sized by Facebook share counts, and the blue/red color borders indicate the political side. Moreover, by visualizing popular online imageboards, they were able to create large scatterplots of images clustered by the similarity of what they portray. Another study by Wu et al. [18] also visualized social media data analytics, whereby they included image data and visualized it using an image cloud to display shared photos, and it shows the most shared images as being larger than the least shared, similarly to a word cloud.

The literature has presented different visualization methods and techniques, highlighting the benefits of using interactive visual analytics and its ability to help users process big data and improve their decision-making in a crisis. Nevertheless, the studies were conducted while considering one source of social media data and providing one to three visualization techniques at once. Furthermore, the existing visualization platforms are limited in terms of integrating multimodal data visualization; therefore, this research proposes integrating different visualization techniques from multiple sources of social media networks into one interactive dashboard that will give the users a broader view of texts, images, video, and audio information in real-time. Multiple data views will give the user different options for solving a problem and choosing the best action plan. Additionally, real-time information is vital to assess and respond to victims in crisis.

## 3. Disaster Management Framework and Need for Information Visualization

**Figure 1:**
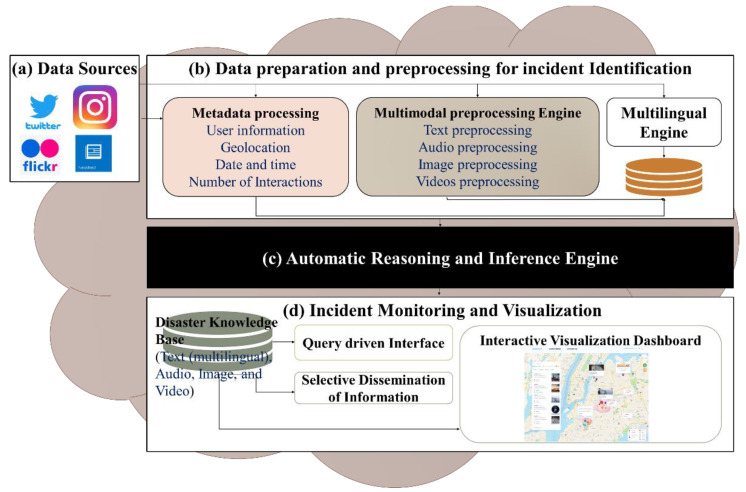
The components of the social media-based incident detection and monitoring system, and the data visualization architecture.

This research paper is motivated by the extension of the social media-based incident detection and monitoring system. Figure 1 presents the proposed architecture of the incident detection and monitoring system that consists of various modules of disaster identification, data collection (shown in Figure 1a), and incident extraction from heterogeneous sources in the multimodal and multilingual format (shown in Figure 1b). Moreover, comprehensive semantic analysis of the multimodal and multilingual data collected in the previous step, through applying artificial intelligence techniques and mapping it with disaster ontology, is presented by the black box in Figure 1c. Efficient representation of the crisis information on the interactive user dashboard for incident-monitoring and visualization to facilitate a prompt emergency response is shown in Figure 1d [8]. The process of data collection and preprocessing involves all steps to prepare it for machine learning and deep learning models of crisis event detection and classification. In this project, we have explored multimodal social media data for crisis event identification, extraction, and classification.

In the vein of textual data processing, the textual information from Twitter has been explored to investigate different topics discussed in disaster-related posts presented in [9]. After applying feature extraction and topic modeling, a pre-trained BERT transformer is used for disaster classification of tweets. In the same project, with a focus on multilingual data, an Arabic dialect identification model was developed based on the BERT algorithm to classify Egyptian, Gulf, Iraqi, Levantine, and Maghrebi dialects by analyzing COVID-19 Arabic conversations on the Twitter network, as published in [6]. For emergency detection and identification from the visuals, in [13], the authors identify emergency needs and responses from visual information to support the humanitarian organizations in reaching out to the affected people and specific locations with their services. An image processing pipeline was used that first inputs an image and applies disaster classification and object detection using a deep learning neural network. The output image was mapped onto the proposed emergency response taxonomy (consisting of emergency response categories and their textual labels). The textual and visual information is classified according to disaster categories such as damage level, affected individuals, caution warning, basic needs requests, and so on. That data helps develop a useful ontology of disasters using the protégé [19] tool to meaningfully integrate this information in order to present it on an interactive dashboard [8].

This research paper mainly focuses on the bottom layer of the architecture for incident monitoring and visualization, as presented in Figure 1d. Many studies emphasized the implementation of disaster information extraction and its classification using artificial intelligence techniques; however, a gap exists in terms of presenting text, images, audio, and video integration on an interactive dashboard to analyze large social media crisis data. The need for such a multimodal dashboard to present such information is highlighted by O’Halloran et al. [20], who have presented various multimodal information solutions in online newspaper articles; therefore, the other layers of the architecture, highlighted in Figure 1a–c, are beyond the scope of this research article. However, we assumed that the disaster information is preprocessed and classified in the previous layers (shown in Figure 1a–c) and used as input for the incident monitoring and visualization layer (shown in Figure 1d) to design and develop the interactive dashboards.

## 4. Materials and Methods

**Figure 2:**
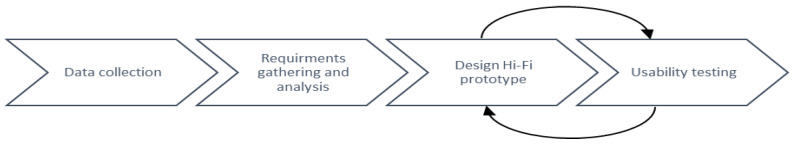
The methodology for developing a multimodal data visualization framework.

This paper aimed to design and evaluate the multimodal interactive dashboard to present disaster-related information. Figure 2 presents the steps of our user-centered design approach in developing the multimodal crisis data dashboards for interested stakeholders. First, we collected the social media multimodal disaster data, requirements from the related literature, and informal surveys of social media platforms. Second, we investigated all the required features and metadata related to social media multimodal disaster-related data. Third, we developed multimodal interactive prototypes using an iterative and incremental approach. Lastly, we evaluated our prototypes for usability using the heuristic evaluation method and improved the prototype based on evaluation feedback.

### 4.1. Data Collection

The social network sites’ (SNS) data used in previous research on EM is largely extricated from Twitter using the Twitter Application Programming Interface (API) [21]. Other than Twitter, many other social media platforms are rich sources of information in terms of text, images, audio, and videos, especially image-based social media such as Flickr and Instagram, which are seen as promising sources of information retrieval in cases of disaster events. Audio-based social media is also gaining more attention and popularity in the form of existing platforms and applications such as Clubhouse and Listen; audio-based social media in the future can be of great use in the case of emergencies and save lives.

Previously, many studies [8,11,12,13] have been conducted in the area of extracting, detecting, and classifying crisis-related information from social media using intelligent models; however, very little attention has been paid to the usable presentation of such information using an interactive dashboard that can provide prompt information to EM teams in various formats and representations from various sources such as text, images, audio, and videos to speed up the emergency response process that results in an excellent user experience.

It is evident that the current user interfaces of EM systems’ dashboards lack usability and user experience [22,23] in terms of multimodal social media information presentation to improve the emergency response. For this purpose, in this paper, we have investigated five dimensions of social media information in our visualization dashboards: text, images, video, audio, and geolocation data. We have selected these five dimensions based on criteria concerning their popularity in user posts, details of the information available through them, and the data available in the previous studies [8,12,13,24]. The fact is that most of the information posted on social media is through the users’ smart devices, which are equipped with cameras and location sensors that make it easier for the users to post on social media in the selected five formats. Data available in the various formats are more informative, and they provide us with more insight into, and details of, an event; however, a balance between represented information and visualization readability should be kept; too much information in the visualization makes it challenging to comprehend [25].

It is impossible to build a readable visualization representing all this data as the amount of data to visualize would be enormous; therefore, data categorizing and abstraction (aggregation) is required for presenting it on the user interface. The extent of data aggregation depends on the aim of a particular visualization; if we want to see all the data of an ongoing emergency, the aggregation period should be minimal (e.g., one minute) [26]. If the data are not urgent, such as data relating to long-term patterns and trends, then the aggregation period can be extended further (e.g., one hour, one day) [26]. Moreover, the selection of visualization techniques depends on the necessary data that is represented; therefore, we are focusing on the most valuable data for EM, which will be presented in the visualizations. For this purpose, the following categorization criteria are proposed: disaster categories, SNS metadata, and SNS multimodal data.

**Figure 3:**
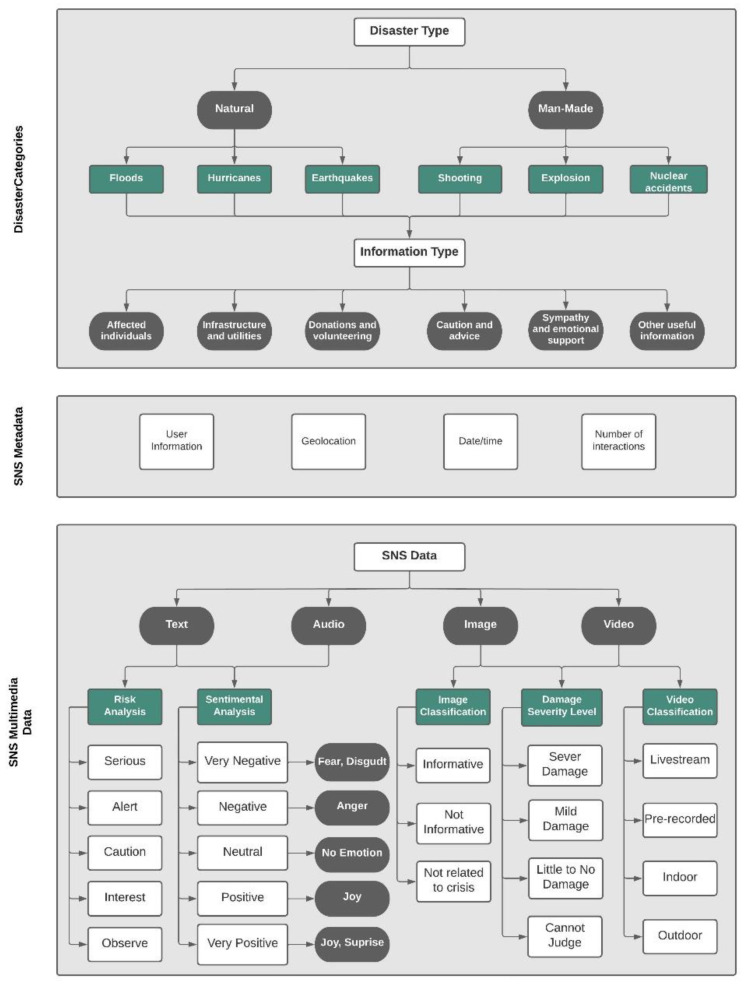
A summary of required disaster-related data collection from social media and their classifications.

For the required collection purposes, we have studied all the data (data and meta-data) details of the Twitter, Facebook, and Flickr social media platforms. Based on the type of data collected from different SNS (Twitter, Facebook, and Flickr), we propose the categorization criteria shown in Figure 3, which illustrates the data types collected and their classifications.

First, in the disaster categories and sub-categories layer, we started with the type of disasters [27]. For this purpose, we gathered and analyzed the types of disasters from various sources, including technical and general papers, online web pages, and internal reports. Consequently, we found that there are mainly two types of disasters, which are natural and man-made. Then, we further categorized the disaster types into information types extracted from [13,28].

Second, in the SNS metadata categories, we gathered the data by exploring several SNS and collecting the data types used in each platform; then, we gathered data types from the developers’ platforms for each SNS. Twitter data was easier to collect, seeing as it is widely used in other studies [15,16,29,30]; therefore, we compared our data with theirs and combined them. We also found that data types from other SNS are similar to the Twitter data collected. Hence, we classified the SNS metadata into four categories: user information, geolocation, date/time, and the number of interactions.

Lastly, we followed the same collection method we used in the SNS metadata layer in the SNS multimodal data categories layer. After collecting the data types found in the SNS, we categorized them into four types: text, image, audio, and video.

#### 4.1.1. Disaster Categories

Categorizing disaster types has been the concern of researchers, governments, and independent agencies. Numerous entities categorize disasters differently and use distinct terminologies. Although researchers mainly follow the same category type as [31], they summarized disaster types into natural and man-made categories, and defined sub-categories for each one. They categorized natural disasters into: (1) “Natural phenomena beneath the Earth’s surface”, such as tsunamis and earthquakes; (2) “Natural phenomena of complex physical origin on the Earth’s surface”, such as landslides; (3) “Metrological/hydrological phenomena”, such as tornadoes, sea surges, and floods; and (4) “Biological phenomena”, such as locust swarms. The man-made sub-categories include: (1) “Conventional”, such as sieges; “Non-conventional warfare”, such as, nuclear disasters; and (2) “Accidents”, such as drowning and explosions.

Each government agency has defined its own disaster categories; however, they often follow the same category pattern. The World Health Organization (WHO) [32] classified disasters into: (1) Natural disasters and (2) Man-made/technological disasters, such as fires, nuclear, and industrial disasters. They further categorized natural disasters into: (1) Meteorological, such as hurricanes; (2) External, such as landslides; (3) Internal, such as earthquakes; and (4) Biological, such as infestations and epidemics. The Federal Emergency Management Agency (FEMA) [27] has developed a website that gives background information on hazards, dangers, and disaster mitigation, and they classified disasters into natural disasters, such as earthquakes and floods, and man-made disasters, such as hazardous materials. We adapted the same disaster categories for our visualization based on the previous research.

**a.**  **Disaster types:**

The first dimension we examine is the type of disaster, starting with two main categories: natural and man-made disasters. Then, after categorizing them further, we have many sub-categories of information types, including accidents, fatalities, landslides, missing people, and wildfires.

- **Natural disaster:** Disasters caused by a major and sudden adverse event from natural causes such as, floods, earthquakes, and hurricanes.
- **Man-made disaster:** Disasters caused by human negligence, error, or harmful intent, such as, shootings, explosions, and nuclear disasters.

**b.**  **Information types:**

The literature that visualized SNS data mainly focused on one category of disasters, such as floods or hurricanes, but other studies identified a broad list of disaster categories and sub-categories. There is an endless list of disaster categories [7,23]; therefore, we have extracted the disaster information type and matched it to each category to create a smaller list. Below, the information types are prepared carefully to include all essential sub-categories.

- **Affected individuals:** deaths, injuries, missing, found, or displaced people, and/or personal updates.
- **Infrastructure and utilities:** buildings, roads, and utilities/services that are damaged, interrupted, restored, or operational.
- **Donations and volunteering:** needs, requests, or offers of money, blood, shelter, supplies, and/or services by volunteers or professionals.
- **Caution and advice:** warnings issued or lifted, as well as guidance and tips.
- **Sympathy and emotional support:** thoughts, prayers, gratitude, sadness, and support.
- **Other useful information:** information not covered by any of the above categories, such as flood level, weather, wind, and visibility.

#### 4.1.2. SNS Metadata

In SNS, with every post sent, there is additional data attached to it. For example, in one Twitter post, in addition to the tweet message, the location, time, date, user ID, the application used, and the device type are all shown within that post. In our visualization, we consider four types of social metadata, which are the most common and essential metadata of SNS.

- **User information:** user handle, display picture, number of followers/following, and number of posts.
- **Geolocation:** profile location, tagger location, mentioned location, geocodes, and geotags.
- **Date and time:** post date and time.
- **Number of Interactions:** Number of replies/comments, retweets and quote-retweets, likes, and views.

To use the SNS metadata in developing a multimodal interactive dashboard, we have assumed the challenges related to disaster metadata, such as its validity, accuracy of location, and justification of trustfulness, which have been solved in the previous layers mentioned in Figure 1a–c. Khatoon et al. [8] reported location-specific keywords and place-based hashtags parsing techniques to improve situational awareness [33], thus crowdsourcing for useful geographic information for tracking location on the map [34]. At this stage, the data is ready to display on the dashboard.

#### 4.1.3. SNS Multimodal Data

Social media users produce various types of social media data. Mainly, we have found text, images, audio, and video data posted by users on social media, which we regard as multimodal data in this paper.

##### Text

Most SNS data is available in textual form; therefore, researchers used automatic tools to analyze a large amount of the textual data extruded. Text analysis is the process of deriving considerable information from textual data. Extracted information from a text can be divided and classified in EM.

**a.**  **Sentimental Analysis**

Understanding people’s emotions can provide insight into how people communicate during an emergency. Emotion detection can provide contextual information for emergency responders; for example, tweets labeled as “fear” might support responders in assessing the mental health of the affected population. Öztürk and Ayvaz [35] used Twitter data to analyze public sentiment towards the Syrian refugee crisis; they collected English and Turkish tweets that included keywords such as “Syrian”, “refugee”, “Suriyeli”, “mülteci”, and “multeci”. To analyze the sentiment score of the English tweets, they used the RSentament package to analyze sentiments at the sentence level; as for the Turkish tweets, they developed a sentiment analysis lexicon. The sentiment score was calculated and divided into five categories: Very Negative, Negative, Neutral, Positive, and Very Positive.

Torkildson et al. [36] analyzed people’s emotions during the Gulf Oil Spill in 2010 by collecting text twitter data and developing a taxonomy of emotion that include eight emotions: “joy”, “anger”, “fear”, “sadness”, “surprise”, “disgust”, “supportive”, and “accusation”. The sentiment score was divided into three categories: Positive, Negative, and Neutral. Table 1 shows an example of the tweets they extracted and the emotions and sentiments labels [36].

**b.**  **Risk Analysis**

Risk analysis of textual data can give insight into the risk level of a disaster occurrence in an emergency; for example, with floods, the risk level in each city zone is different. Kwon and Kang [15] analyzed the risk level of a tweet’s text and the vulnerability of the Twitter location, then, they defined the data using a 5-by-5 risk evaluation matrix, and the result value was classified into five levels from A (Serious), B (Alert), C (Caution), D (Interest), and E (Observe). The risk level of a tweet’s text depends on the level of the keyword, disaster sign word, and adverbs. For example, if a tweet included keywords such as “Heavy rain”, “torrential rain”, and “downpour” only, then it would be considered as being a level two risk expression.

##### Audio

Audio data has been around for a long time; SNS provide users the option of sending voice messages instead of texting, and many prefer audio because it sends the message and conveys what the user is feeling at that particular moment. We can extract meaning and information from a person’s voice during an emergency by analyzing audio. Studies that include audio data mainly focused on emotional and sentimental analysis, identifying multiple acoustic features such as pitch, speech intensity, bandwidth, and audio length. Poria et al. [37] stated that an analysis done by Scherer [38] indicated that humans could recognize emotions from speech about 60% of the time; it also showed that sadness and anger are the easiest emotions to detect. Moreover, Caridakis et al. [39] analysis showed a 93.30% accuracy in identifying anger and 76.67% in identifying sadness.

For visualizing audio data, we classified the data based on the same sentimental analysis, as explained previously.

##### Images

Before the rise of social media, automated image recognition and object detection had been studied for a long time. Despite comprehensive research focusing primarily on social media textual data and visualizing text and location, there has been little work on visualizing images to improve emergency response. There exist a few disaster image datasets, such as Natural Disaster Events by Alam et al. [12] and Asif et al. [13], and Fire images by Daly and Thom [40]. These datasets were collected from several sites such as Twitter and Flickr, but none of them actually visualize the images; they only show the temporal distribution of messages that include the images. We visualized the images by adopting some of the classifications to overcome this limitation, and annotations were made in these datasets.

**a.**  **Images Classification**

During an emergency, thousands of posts are generated, and thus, determining whether a post contains critical information that is useful for emergency responders or not is important to reduce information overload. The studies by Olteanu et al. [28], Alam et al. [41], and Peters and De Albuquerque [42] classified their data collected from SNS into informative, not informative, and do not know or cannot judge. Classifying images into two main categories will help reduce the search time.

○ **Informative:** If the image is useful to emergency responders, where it is related to the crisis, shows the affected area, and helps them understand the situation. For example, images of floods, fire, and hurt people.
○ **Not Informative:** If the image is related to the crisis but not too useful to emergency responders in terms of helping them understand the situation. For example, images that are trolling and off-topic, or based on rumors, and humor.
○ **Not related to crisis:** Posts that include advertisements or memes.

**b.**  **Damage Severity Level**

A single image can convey more information than 280-character text. From an image, we can precisely see the level of damage that happened to a road or bridge. The damage severity was classified into four categories [41], although previous studies only considered the physical damages (i.e., flooded roads); nevertheless, in our research, we considered non-physical damage (i.e., smoke from the fire) as well.

**Figure 4:**
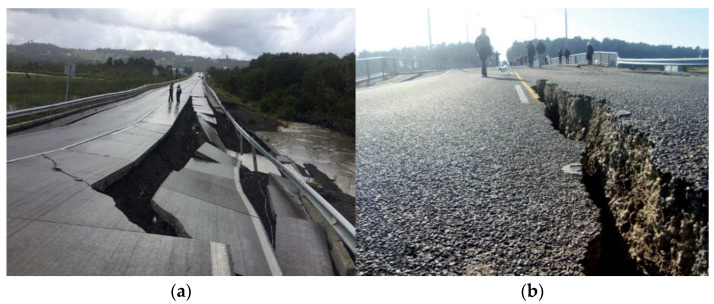
(**a**) Depicts a severely damaged bridge after an earthquake in New Zealand. (**b**) Depicts a mildly damaged bridge after an earthquake in Chile.

○ **Severe damage:** Includes images that show considerable destruction of a foundation. For example, damaged roads, non-crossable bridges, or big clouds of smoke.
○ **Mild damage:** Images of partly damaged roads, buildings, or houses. For example, if part of a road needs to be blocked off but is still usable. Figure 4 shows a comparison between (a) a severely damaged bridge after a major earthquake and (b) a mildly damaged one [43,44].
○ **Little to no damage:** Images of damage-free foundations or ones that have very little damage. For example, wall cracks in building due to age.
○ **Cannot judge:** Includes images that are of low quality.

##### Videos

Videos and images contribute to 80% of unstructured data [45]. Nowadays, closed-circuit television (CCTV) cameras, also known as video surveillance, are ubiquitous in streets, stores, and homes. Most videos that are captured and typically broadcasted are sent to a small (closed) number of monitors in the C&CC. SNS for live streaming, such as YouTube and Twitch, are also helpful during an emergency; people begin live streams from the center of the crisis that helps emergency responders to assess the situation. Video contains significantly more information than an image, and there have been multiple studies that developed video datasets; however, there have been none that are only disaster-related. Moreover, there has been no visualization of video data found. In a study by Shullani et al. [46], they developed a VISION dataset that includes video and images for multimedia forensics. The authors organized the video data into nine categories; we adopted some of their video classifications, in addition to the image classification discussed in the previous section. We have adapted image classifications because videos are a collection of images that enable visualizing the video data in a meaningful way to improve the effectiveness and efficiency of EM.

**a.**  **Videos Classification**

Videos are a collection of images that make them larger in size; therefore, it takes the network and the server more time to process. To make the page load faster, we first classified the video data into live and pre-recorded videos; then, we classified them into indoor or outdoor videos.

○ **Livestream:** Livestream videos are videos recorded and broadcasted in real-time, and they are built for engagement. When a person starts a livestream, there are other people commenting live on the video, and within the comments, there may be helpful information.
○ **Pre-recorded:** Videos that are recorded in advance and then shared in SNS.
○ **Indoor:** Includes videos that are taken indoors, such as offices, houses, or stores.
○ **Outdoor:** Includes videos that are taken outdoors in open areas, such as streets, gardens, or open markets.

### 4.2. Requirments Gathering and Analysis for Multimodal Dashboard of Crisis Information

Before starting our visualization design, we needed to gather the technical and user requirements. We first discussed the different tools available for designing prototypes, and then we discussed which tool enables us to design the prototype based on all the data gathered. Lastly, we collected a list of user requirements from different studies and added new requirements that applied to our study.

#### 4.2.1. Technical Requirements

Many data visualization technologies [40] make creating visual representations of big datasets easier for data visualization designers. When working with datasets that include hundreds of thousands or millions of data points, automating the visualization process makes a designer’s job much easier, at least in part. Tableau [47] is one of the most popular visualization tools, and it is a good option for creating maps, in addition to different types of charts to visualize textual data. Another popular tool is Microsoft Power BI [48] which offers access to massive data sources and data visualization templates, and is compatible with other Microsoft office products; however, after going through the popular data visualization tools, we found that only power BI has the feature of visualizing image data through image grids. Although these visualization technologies help visualize big data, they do not offer options to visualize images, videos, and audio; therefore, we used a wireframing and prototyping tool to show a complete view of our suggested system.

Various prototyping tools have different features for complex design goals, such as Axure [49], Sketch [50], InVision [51], and Figma [52], with empowering user interfaces and user experience (UI/UX) designers to create high-fidelity prototypes rapidly. One of the most well-known and widely used is Adobe Experience Design (Adobe XD) [53]. It provides many features and options for creating wireframes and interactive high-fidelity prototypes, such as adding screens, shapes, buttons, animations, and simple click interactions that can be shared with developers to help them in the development phase. Using Adobe XD, we designed a click-through high-fidelity prototype to demonstrate the minimum interaction between pages.

#### 4.2.2. User Requirements

We extracted several user requirements from [13,45,46] that are relevant to the user interface and visualizations design in order to design our multimodal crisis management visualization. Each study showed unique and similar requirements to be met; hence, in Table 2, we exhibit a list of functional user requirements that are grouped based on relevance.

Additionally, we have also defined non-functional requirements (usability attributes). The non-functional attributes include: (a) memorability; (b) satisfaction; (c) efficiency; (d) effectiveness; and (e) error handling. We also took the variety of charts, visual consistency, and customization options into account to ensure that the visualization is adaptable for each type of crisis and is not dependent on the nature of the events.

### 4.3. Interface Design Process

The development of the multimodal data visualization dashboard followed a user-centered, iterative process that includes the following steps: (1) reviewing the literature and similar crisis management systems; (2) requirements gathering and analysis; (3) data collection and classification and the interactions required; (4) designing the dashboard; (5) usability evaluation and improvements.

The design consists of both functional and non-functional requirements that we gathered. We selected and proposed visualization elements incorporated in the UI based on these requirements, such as image network and sentimental audio analysis. Furthermore, the required interactions from the visualizations were determined using an established visual analytics model, which states that visual information should be designed to provide “Overview first, zoom after, details on demand” [56].

**Figure 5:**
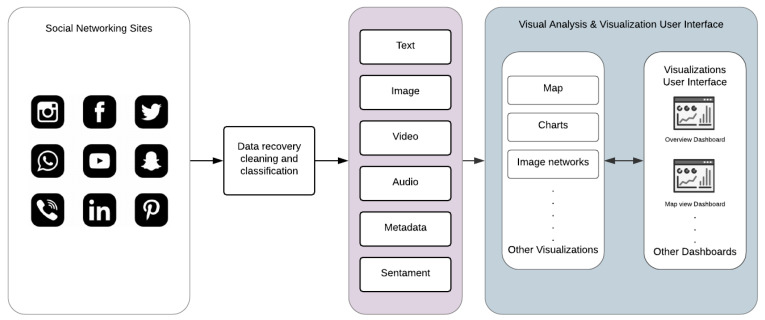
Multimodal data visualization framework.

The framework shown in Figure 5 is composed of three different layers: (a) information recovery, (b) visual analysis elements, and (c) the visualization user interface; they work independently and interact with each other to deliver the desired output [16,55]. 

After the data is retrieved, cleaned, and classified, visual analytic techniques will be applied to present a visual representation to the end-user. Each data type is represented using a different interactive visual element. In addition, some new visual analytical elements have been designed to facilitate datatypes that have never been visualized and are not supported in known visualization tools, as discussed in Section 4.1. This framework enables easy integration of the custom visual analytic elements needed according to the end user’s requirements and type of crisis events. We have considered the preprocessed, clean, and classified disaster data (text, image, audio, and video) reported in the previous research articles [6,8,9,13] to develop the interactive dashboard.

## 5. Interactive Dashboard

**Figure 6:**
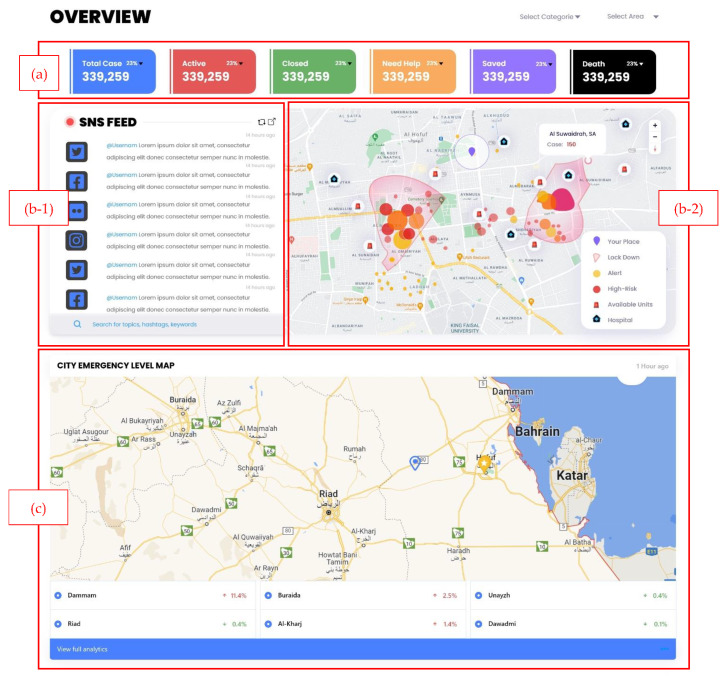
Multi-monitor visual analytics elements. (**a**) Total case statistics, (**b-1**) live SNS feed, (**b-2**) heatmap, and (**c**) city emergency level map.

**Figure 7:**
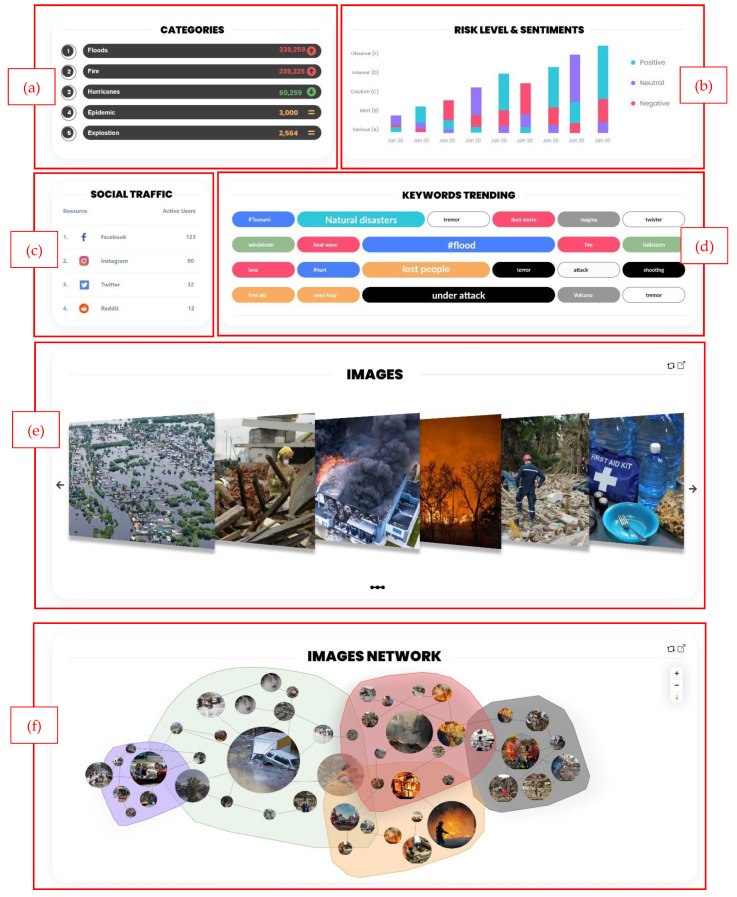
Multi-monitor visual analytics elements. (**a**) Crisis categories ranking, (**b**) risk and sentimentally levels bar chart, (**c**) social traffic ranking, (**d**) keywords word cloud, (**e**) image gallery, and (**f**) image network.

**Figure 8:**
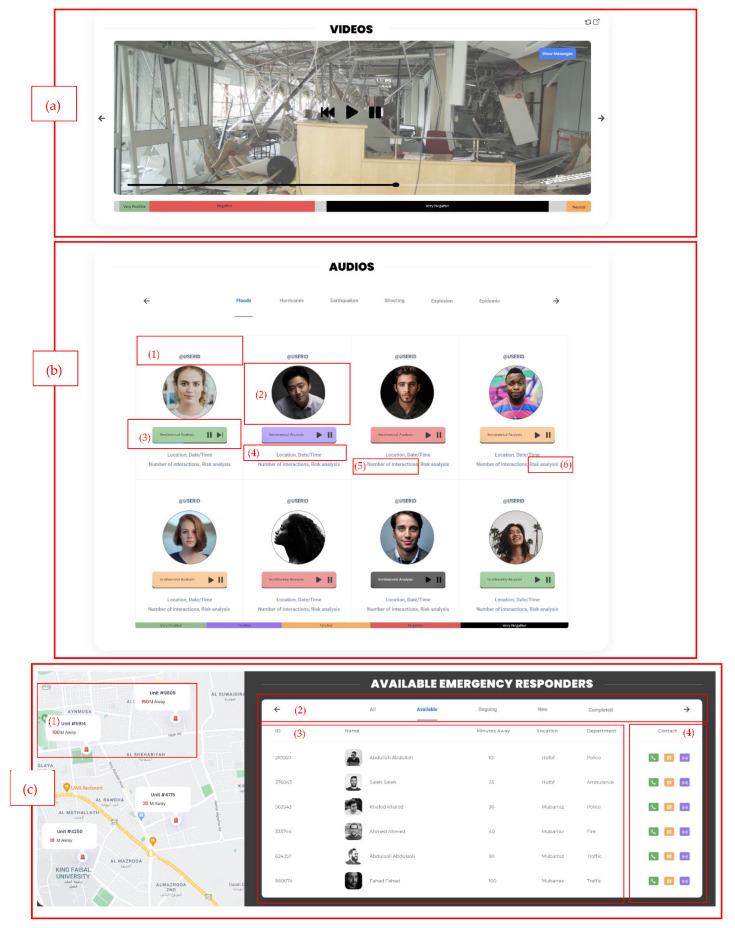
Multi-monitor visual analytics elements. (**a**) Video sentiment analysis. (**b**) audio map which includes: (1) username, (2) user display picture, (3) audio player, (4) metadata, (5) number of interactions, and (6) the risk analysis of the audio. (**c**) collaboration board that includes: (1) a map, (2) the board where the users can be categorized, (3) the emergency units’ information, and (4) the contact buttons.

This research presents high-fidelity prototypes of the visualizations using the Adobe XD [53] program that includes synthetic social networking sites’ (SNS) multimodal data. Moreover, our visualization supports collecting data from multiple SNS platforms. We concentrated on building static visualizations that represent crisis-related datasets that are classified based on datatype. Dynamic visualizations are possible in the future when receiving data in real-time. The visual analytics are illustrated through two primary dashboards: (a) multi-monitor flow and (b) one-page flow. Both dashboards have the same heading that includes: (i)the current date/time; (ii) search bar; (iii) notification center; and (iv) user profile. The multi-monitor flow consists of 12 visual analytic elements listed in Table 3, and the visualization UIs (User Interfaces) are illustrated in Figure 6, Figure 7 and Figure 8.

**Figure 9:**
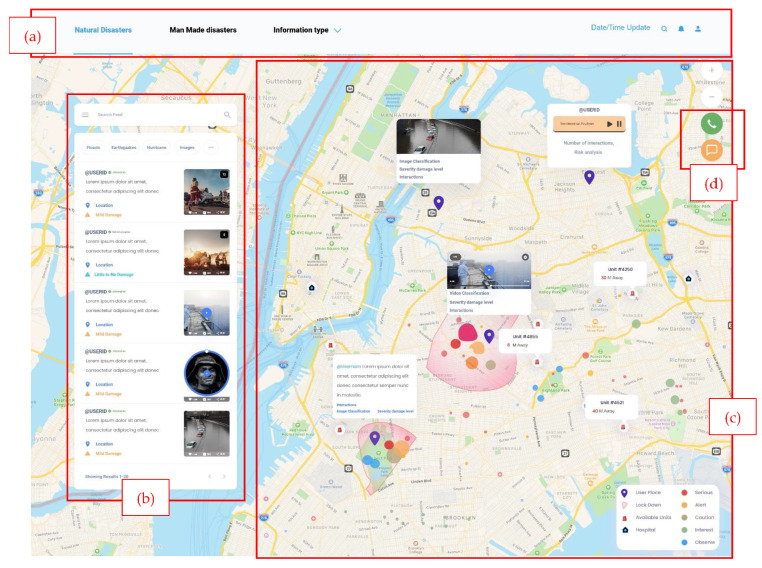
One-page flow visualization user interface of text, image, audio, and video disaster information. (**a**) the header and disaster types, (**b**) the live SNS feed is shown on the map, (**c**) user’s location on the map, and (**d**) call and message buttons.

On the other hand, the one-page flow shown in Figure 9 consists of one main visual analytics element: the map. This dashboard is designed as an alternative for users who work on a small screen and need preliminary information. In Figure 9a, the header and disaster types are shown (natural or man-made). In Figure 9b, the live SNS feed is shown on the map and includes the username, message, metadata, risk level, and any attached image, video, or audio. The user can search and filter based on disaster categories. When the user clicks on a message, it can be displayed on Figure 9c, the map highlighting the user’s location. Furthermore, in Figure 9d, call and message buttons are shown for the emergency operators to contact the closest emergency responder.

The visualization UIs were designed based on the end-user requirements, displaying the most critical information immediately when a user views the dashboard. As mentioned earlier, the user can view more additional information, such as individual statistics or alternative interpretations of the data, such as image and video classifications and damage severity level, which are available with a few clicks. All visualizations are interactive and offer zoom-in/zoom-out options, they include error messages, as well as help buttons to show descriptions to promote ease of use.

The usability of our framework visualization user interface was tested using the heuristic testing method [23]. Although this testing method requires having a small set of evaluators, ideally between three to five, with three evaluators, 75% of the major problems of the interface can be indicated [58,59]. With that being the case, to conduct this test, three evaluators with IT backgrounds and usability testing experience were recruited to examine the interface and judge its compliance with the heuristics. In a controlled environment, each evaluator was asked to inspect the two dashboard views that are shown in Figure 6, Figure 7, Figure 8 and Figure 9 alone to ensure an unbiased evaluation. After all the evaluators have completed their evaluations and documented the severity rating of each heuristic principle following the 0 to 4 rating scale, with 0 representing no usability problems and 4 representing usability catastrophes, as well as documenting their recommendation or violations found, only then can they communicate to discuss their findings and aggregate them.

After combining the results and ratings into Table 4, we found two cosmetic problems, one minor usability problem, and nine heuristics which were not violated. A summary of the negative findings is presented as well. Overall, the evaluator’s evaluation showed positive feedback with minor alterations to be made. We also took into account their recommendations and updated the visualization.

## 6. Discussion and Conclusions

This paper presented the comprehensive requirements for multimodal social media disaster-related interactive dashboards. The data, in various formats, are integrated and have been utilized to develop interactive dashboards using iterative prototyping techniques. The evaluation of proposed multimodal interactive dashboards reported above-average usability. The results are exceptionally useful in developing multimodal dashboards for disaster emergency services as well as multimodal dashboards in general that may use a similar type of information.

Presenting multimodal massive social media data on an interactive dashboard is a challenging task, and in this sense, previous studies have made limited contributions. This paper filled this gap by exploring the designs that include multiple visualization techniques to support SNS multimodal data, and this study presented them in a user-friendly manner. By comparing our designs with those of other disaster management visual interfaces, we can see that geolocation data is visualized using similar techniques. The study in Onorati et al. [16] used density maps to analyze where users gather during a disaster; similarly, in our design, we used a density map, but added more details to help emergency operators in their decision-making. For image data, Bhargava et al. [17] proposed visualizing them using a treemap, but it did not precisely present the images, and there was no other information extracted except for the number of interactions; therefore, we visualized them using two techniques. First, we used the image gallery, to give a much clearer for of the images and the number of interactions with each. Moreover, the image network shows the relationships between images, from where they spread, and what sentiments they associate with them. For video and audio data, we found no literature or tools that visualized these data types, so we took emergency operators of such data types into consideration. Therefore, we suggested first giving an overview of the user sentiment—this will help emergency operators classify urgent cases—then, when more information is needed, they can view a Gantt chart of how user sentiment changes. The proposed multimodal interactive dashboards are not only used to present the social media disaster information, but they can be expanded to other domains confronting similar challenges of massive multimodal data.

Various EM systems are available that take data from social media and display disaster information using a dashboard. Senseplace2 [29] is an EM system that uses textual data from Twitter and displays it using a map, color grids, and graduated circles to display the frequency and location of the tweets and the time interval. The display of Senseplace2 can be expanded by employing Figure 7a,b to categorize the tweets for their frequencies and improve timeline presentation. In addition, the tweets’ presentation can be enhanced by highlighting the related hashtags using Figure 7d. AIDR (Artificial Intelligence for Digital Response) [60] is specialized in collecting and preparing multimodal (text and images) disaster information from social media channels. They use an AIDR dashboard to display real-time social media disaster data collection from various locations worldwide, which summarizes their data in the offline collection, total feeds, running collections, and total collection. Given the richness of the data, in terms of text and images related to disaster events worldwide, our proposed dashboard in Figure 6, Figure 7 and Figure 9 is suitable for presenting disaster information that is preprocessed and classified using artificial intelligence techniques. Ushahidi [24] is a famous open-source EM platform that allows disaster data gathering from many sources such as email, Twitter, the web, and SMS. The disaster information is displayed using maps, charts, graphs, timelines, and color visualization. The dashboards proposed in Figure 6a–c are highly aligned with the disaster data supported by the Ushahidi platform and can be helpful in the visualization of emergency data in a usable and effective manner. Currently, we have highlighted some specific scenarios of existing EM systems that can be improved by employing our proposed disaster dashboards. In future, these designs can be integrated with existing EM systems such as Ushahidi to study their effectiveness in an emergency response.

This paper mainly focuses on input data as the social media posts are related to disaster or crisis information. Furthermore, the dashboard was designed by keeping in mind the stages of disaster (stage 2, the storm; stage 3, the peak; stage 4, the plateau; stage 5, the decline [5]); however, these dashboards can be expanded to different scenarios that focus on emergency information elicitation from other sources such as satellites, citizen emergency reporting portals, and organization information systems. The application and implications of an interactive disaster dashboard can be endorsed with the help of one of the crucial scenarios of a hospital evaluation. Rambha et al. [61] highlighted the challenge of evacuating vulnerable populations, such as patients, from the hospital during hurricanes. Sahebi et al. [62] explored the factors affecting a hospital patient’s evacuation process during a fire and planning their evacuation in the times of disease outbreak [63]. The dashboards presented in Figure 6a,c are suitable for presenting qualitative data in terms of hospitals’ capacities, patients’ statistics, and resources such as vehicles and beds statics, and the heatmap in Figure 6b-2 can highlight high-risk locations. The hospital evacuation-related information can be optimally displayed to the emergency workforces to keep them informed and motivated to continue their emergency operation. It is worth noting that we have discussed the applicability of our proposed design to potential emergency scenarios. In the future, it is a significant area of interest to investigate the pros of deploying interactive emergency dashboards on similar EM systems.

In the future, we aim to integrate our multimodal interactive visualization dashboards, designed with our EM systems [8], to support EDM. Currently, we have evaluated the proposed designs based on heuristic evaluation; however, the functional prototype will be evaluated using the other usability evaluation methods such as usability testing or via a cognitive walk-through. Cleland et al. [64] highlighted the importance of involving diverse users and domain experts in the usability evaluation of big data analytic platforms in finding the most critical problems and root causes for issues. Motivated by the previous studies, we aimed to conduct the usability evaluation by involving the domain experts, integrating the dashboards with a fully functional EM system, and involving the system’s real users. Moreover, the number of dimensions represented in the visualization will probably grow (e.g., the number of available units, activities recognized from the accelerometer data, and statistics of emergencies can also be included in the visualization). Furthermore, these designs will be adapted to other domains and evaluated in terms of suitability to make them available to substantial stakeholders. 

## Figures and Tables

**Table 1:** Example tweets coded for emotion and sentiments.

SN	Tweet Text	Sentiment Label	Emotion Label
1.	Coolant oil spill, leak from CNC machine #Oil #Oilleak #Oilspill #Coolantoil #CoolantOilbecomeyellowcolor	Neutral.	No emotion.
2.	Cheering myself up by listening to a podcast about the Exxon Valdez #oilspill that ruined the pristine waters of Alaska. 😕 #environment	Negative.	Disgust.
3.	Great to work with Dr Colin Wood and Caleb Karmelich to promote their #OilSpill response technology. By quickly removing oil from seawater (even at low concentrations), their tech could make the clean-up process faster, cheaper, and more efficient. [URL]	Positive.	Supportive.
4.	Humanity needs to rethink its relationship with fossil fuel. #sustainability #BP #oilspill	Negative.	Accusation.
5.	#Trumo administration unravels offshore #safety regs. 11 men perished in the #BP #DeepwaterHorizon #oilspill disaster and the administration	Negative.	Fear.

**Table 2:** High-level user requirements of disaster interaction dashboard.

Requirements	Description
(R1)—User friendly interface [54,55]	The visualization user interface is easy to learn and use.
(R2)—Interactive visualization [54,55]	The visualization gives the user the option to tailor the interface to their needs and to control how information is visually represented.
(R3)—Real-time visualization [54]	The visualization presents information in real-time for immediate or future actions. The information needed to carry out actions and plan strategies must be presented clearly and precisely.
(R4)—Visualization of multimodal data	The visualization includes four main data types (text, image, video, and audio), along with the metadata (i.e., time, date, location).
(R5)—Visualization of geographical data [16,55]	The visualization includes and interactive map to show where the social activities were posted from.
(R6)—Visualization of sentimental data	The visualization shows the sentiments associated with the collected posts.
(R6)—Topic identification [16]	The visualization highlights the most discussed topics according to the number of interactions.
(R7)—Topic search [16]	The visualization allows the user to search for messages that were posted about a specific event.
(R8)—Collaboration [54]	The visualization enables collaboration and communication between users.

**Table 3:** Multi-monitor visual analytic elements of multimodal information.

	Visual Analytics Element	Figure	Purpose
1	Total case statistics	Figure 6a	To give the user a full view of total cases and how many are still active, closed, need help, saved, and dead.
2	Live SNS feed	Figure 6b-1	The SNS live feed will show messages from different SNS platforms and include the most recent/ most interacted messages. The user can search for a specific topic, hashtag, or keyword. Additionally, the user can filter based on category. Moreover, when the user clicks on one of the messages in the feed, the location from which that message has been posted will be highlighted on the map.
3	Heatmap	Figure 6b-2	The heatmap will display the message locations, lockdown areas, and areas that are high risk or on alert. Moreover, the available units will be shown on the map.
3	City emergency level map	Figure 6c	The risk level for main cities will be shown, and it will indicate the increase or decrease of risk level in each city. Moreover, the user can zoom in/zoom out on the map and select a specific city to show its risk level.
4	Crisis categories ranking	Figure 7a	The ranking of the crisis categories is based on their occurrence. The element will show whether there are increased, decreased, or no changes in crisis occurrence. The user can filter by day, week, month, or year.
5	Risk and sentiments levels bar chart	Figure 7b	The bar chart y-axis shows the risk level (A), (B), (C), (D), (E), and the x-axis shows the dates of crisis occurrence. The color of the bars indicates the percentage of positive, neutral, and negative sentiments. The user can filter by date and risk level.
6	SNS traffic ranking	Figure 7c	This element will show which SNS is most used during a crisis and how many active users are posting at that time.
7	Keywords word cloud	Figure 7d	The frequency of each keyword/hashtag/topic is represented by a proportional font size, and related words are illustrated with the same color.
8	Image gallery	Figure 7e	This element illustrates a group of images that have a spike in the number of interactions and will be displayed in the order from highest to lowest, and the images can be filtered by category and date.
9	Image Network	Figure 7f	Network visualization examines the relationships between entities. In our proposed image network, we group the images based on the sentiment gathered from each image or the text associated with it. The importance of an image is represented by its size. Additionally, the lines between images represent the relation of one photo to another. For example, if a user tweeted a photo and then added another photo to the thread, these two images would be connected in the visual.
10	Video sentiment analysis	Figure 8a	Similar to the image gallery, the video would include a sentimental analysis while watching the video. In addition, the message and metadata associated with the video can be displayed when enabled.
11	Audio map	Figure 8b	This can be viewed as being similar to a treemap. First, the audio data are divided into categories. Second, each audio file will include: (1) username; (2) user display picture (the photos included in the design are obtained from Unsplash websites that provides copyright free stock photos [57]); (3) audio player (colored according to the sentiment analysis of that audio); (4) metadata (location/time/date); (5) number of interactions, which will be how the audio files are arranged; and (6) the risk analysis of the audio.
12	Collaboration board	Figure 8c	This element shows: (1) a map of emergency unit locations; (2) the board where the users can be categorized based on their availability, in terms of ongoing (already on a case), and completed cases; (3) the ID, name, location, distance from the crisis location, and department are listed; and (4) the user can contact, message, or alert other users.

**Table 4:** Quantitative and qualitative results of the heuristic evaluation of proposed prototypes.

Heuristics	Severity Rating	Violation	Recommendations
Visibility of system status	2	No return button to the home page from the contact page.	Add a BACK button to the home page.
Match between the real world and system	0	No violations found.	No improvements are needed.
User customizing, control, and freedom	1	No page path is clear to the user that shows previously visited pages.	The project is still in the prototype phase. In the future, the URL will show the page path.
Consistency and standards	0	No violations found.	No improvements are needed.
Error prevention	1	Missing confirmation message for some actions.	There should be a confirmation message on the contact page after pressing call or message icons to ask users if they are sure to complete the process.
Minimizing user memory load	0	No violations found.	No improvements are needed.
Flexibility and efficiency of use	0	No violations found.	No improvements are needed.
Information representation mode	0	No violations found.	Adding an information symbol on each visualization to clearly explain the purpose of each section.
Help users recognize, diagnose, and recover from the error	0	No violations found.	No improvements are needed.
Spatial organization and perspective	0	No violations found.	No improvements are needed.
Data reduction and manipulation	0	No violations found.	No improvements are needed.
Control the published content	0	No violations found.	No improvements are needed.

## Data Availability

Not applicable.
